# Does Gesture Lighten the Load? The Case of Verbal Analogies

**DOI:** 10.3389/fpsyg.2020.571109

**Published:** 2020-09-17

**Authors:** Acacia L. Overoye, Margaret Wilson

**Affiliations:** ^1^Behavioral Science Department, Utah Valley University, Orem, UT, United States; ^2^Psychology Department, University of California Santa Cruz, Santa Cruz, CA, United States

**Keywords:** gesture, working memory, cognitive load, offloading, problem solving

## Abstract

Gesturing has been shown to relay benefits to speakers and listeners alike. Speakers, for instance, may be able to reduce their working memory load through gesture. Studies with children and adults have demonstrated that gesturing while describing how to solve a problem can help to save cognitive resources related to that explanation, allowing them to be allocated to a secondary task. The majority of research in this area focuses on procedural mathematical problem solving; however, the present study examines how gesture interacts with working memory load during a verbal reasoning task: verbal analogies. Unlike previous findings which report improved performance on secondary tasks while gesturing during a primary task, our results show that participants showed better performance in a secondary memory task when being prohibited from gesturing during their explanation of verbal analogies compared to being allowed to gesture. These results suggest that the relationship between gesture and working memory may be more nuanced, with the type of task and gestures produced influencing how gestures interact with working memory load.

## Introduction

People spontaneously produce hand movements, gestures, alongside speech. The use of gesture is cross-cultural and individuals from different backgrounds produce gestures tied to their cultural and linguistic heritage (Kendon, 1995; Kita, 2009). The gestures speakers produce are not mere hand-waving but confer benefits to listeners and speakers alike (Novack and Goldin-Meadow, 2015; Dargue et al., 2019). Gesturing while speaking has been found to facilitate problem solving (Cook and Tanenhaus, 2009; Beilock and Goldin-Meadow, 2010; Chu and Kita, 2011; Eielts et al., 2018), learning and memory (Stevanoni and Salmon, 2005; Broaders et al., 2007; Goldin-Meadow et al., 2009; Stieff et al., 2016), and speech production and organization (Graham and Heywood, 1975; Rauscher et al., 1996; Morsella and Krauss, 2004; Hostetter et al., 2007; Jenkins et al., 2017). Gesture has also been shown to improve comprehension, and this enhancement extends across age groups (Dargue et al., 2019). Some have suggested that the beneficial effects of gesture on problem solving and learning are related to how gesture can assist in managing working memory load (Goldin-Meadow and Wagner, 2005; Goldin-Meadow, 2011).

Individual differences in working memory can influence the relationship between gesture use and comprehension. Individuals with lower visuospatial and verbal working memory capacity have been found to produce co-speech gestures more frequently (Chu et al., 2014; Gillespie et al., 2014; Pouw et al., 2016). On the side of comprehension, individuals appear to be more sensitive to information conveyed in gesture when they have higher visuospatial working memory capacity (Wu and Coulson, 2014a,b; Özer and Göksun, 2019). Not only is the extent to which an individual produces gesture and their sensitivity to gesture influenced by their working memory capacity, research has also shown that the production of gesture can change how an individual uses working memory.

Goldin-Meadow et al. (2001) studied the relationship between gesture production and working memory load in a dual-task paradigm with both children and adults. Adults were given a primary task of solving and explaining math problems [factoring polynomials such as x^2^ + 4x + 4 = () ()], while completing a secondary memory task of remembering letters. In each trial of Goldin-Meadow et al. study, participants first solved a factoring problem and were then presented with letters to remember. Participants then explained how they solved the factoring problem and were either permitted to move their hands or required to keep them still while speaking. Finally, participants recalled the set of letters. Results showed that when the participants gestured during their explanation, they subsequently recalled the letters more accurately compared to explanations where they did not gesture. Goldin-Meadow et al. (2001) explained that gesturing reduced working memory load during explanations, resulting in a greater allocation of cognitive resources to the maintenance of letters in working memory for the secondary task. Further research has demonstrated that co-speech gestures manage working memory load more effectively than meaningless hand-waving (Cook et al., 2012), in visuospatial working memory tasks (Wagner et al., 2004), and when gestures refer to problems that are not in the present environment (Ping and Goldin-Meadow, 2010). Additionally, the production of co-speech gestures during explanations appears to be especially effective at reducing working memory load for individuals with low working memory capacity (Marstaller and Burianová, 2013).

There are several theoretical explanations for how gesture reduces working memory load. Producing gestures may help speakers to simulate visuospatial and motoric representations more easily, thereby freeing up additional resources that would have otherwise been necessary for creating simulations (Hostetter and Alibali, 2008; Ping and Goldin-Meadow, 2010; Risko and Gilbert, 2016). Alternatively, the production of gesture may provide speakers with externalized frameworks for problem solving and assist in reducing load by chunking mental work into manageable units (Kita, 2000). Gesturing may also help a speaker shift load from verbal working memory to other, visuospatial, or motoric representations (Paas and Sweller, 2012).

While these studies suggest that gesture alleviates working memory load during speech, much of the dual-task research on gesture and working memory load has focused on mathematical problem solving (Goldin-Meadow et al., 2001; Wagner et al., 2004; Cook et al., 2012; Marstaller and Burianová, 2013). There are several reasons to question whether the relationship between gesture and working memory load found in mathematical problem solving will generalize to other types of problem solving. First, finding and explaining the solution to math problems is often a procedural process. Consider factoring problems: although the numbers in the polynomial vary, the steps a problem solver goes through to factor the polynomial are consistent across problems. This property of factoring problems makes them particularly well suited to benefit from gestures. The gestures produced during the explanation of a factoring problem serve as a repeated structural hangar for speech, saving cognitive resources for a secondary task. In other types of problem solving, gestures may be germane to the specific contents of each problem. Instead of reinforcing a repeated procedure, gestures in other types of problem solving may illustrate unique relationships or surface level details that change across problems.

A second point is that different tasks may elicit different types of gestures, and these may interact with working memory in unique ways. The gestures produced by speakers while explaining factoring problems are primarily deictic (see Wagner et al., 2004; for examples) and the working memory load reduction observed for the speaker may be a result of linking information in speech to representations in the present environment (Ping and Goldin-Meadow, 2010). Although this point was addressed in Ping and Goldin-Meadow (2010) where it was found that iconic gestures produced during explanations of conservation problems about non-present objects can reduce working memory load for children, it is an open question whether the same holds true for adults and for other tasks which elicit different types of gesture. As other work has found that the gestures speakers produce during other types of reasoning tasks are associated with individual differences in working memory (Chu et al., 2014), it seems important to investigate whether the various gesture elicitation tasks result in different effects of gesture production on working memory load.

Finally, the math problems used in previous research rely on numbers – symbols that do not necessarily have strong visuospatial features that could potentially mislead problem solvers. When explaining a math problem, gestures can index numbers and how they relate to each other with less of a chance to introduce irrelevant information into solving the problem. Previous research has found that for certain problem-solving tasks (analogical problem solving, Tower of Hanoi), gestures can interfere with coming to a correct solution because they introduce irrelevant information (Trofatter et al., 2015; Hostetter et al., 2016).

In the present study, we adopt the dual-task paradigm recruited in previous research (e.g., Goldin-Meadow et al., 2001; Wagner et al., 2004; Cook et al., 2012) and investigate whether a different primary task, verbal analogies, influences the relationship between gesture and working memory load. One reason for choosing analogies is that simply, verbal analogies are a different type of task than the spatial and mathematical problem-solving tasks that are often used in this type of work. Gestures can represent analogical relationships (Cooperrider and Goldin-Meadow, 2017) and have the potential to encourage a variety of different iconic, deictic, and beat gestures. Further, there is no set procedural formula to solve an analogy and they require the problem solver to consider non-abstract information.

## Experiment 1A

Experiment 1a used a dual-task paradigm to investigate the influence of gesture production during a primary verbal analogy task on a secondary memory task. Similar to previous gesture and working memory work using this paradigm (e.g., Goldin-Meadow et al., 2001; Wagner et al., 2004; Cook et al., 2012), performance on the secondary memory task served as measure of working memory load. Better performance on the secondary memory task when producing gestures as opposed to being prohibited from gesture during the verbal analogy task would indicate that gestures assist in alleviating working memory load. Alternatively, improved performance on the secondary memory task while being prohibited from gesturing during the verbal analogy task would indicate that producing gestures does not assist in reducing working memory load for the analogy task.

### Method

#### Participants

Forty-four undergraduates from the University of California, Santa Cruz (UCSC) participated for partial course credit. Participants were recruited from psychology courses at UCSC through the Sona Systems subject pool and were required to be native speakers of English to be eligible for the study. Four participants were removed from the analysis because they did not write down responses to the task or did not complete the experiment. The study was reviewed and approved by the UCSC IRB. The participants provided their written informed consent to participate in this study.

#### Design

The experiment used a within-subjects design with gesture instruction as the independent variable (gesture encouraged vs. gesture prohibited) and performance on the secondary memory task as the dependent variable.

#### Materials

Forty verbal analogies were selected for use in the study. All analogies followed the form, “A is to B as C is to …” such as “Hat is to head as roof is to …” Analogies were written such that they relied on many different types of relationships between analogous items such as color, shape, movement, and spatial relationships. Analogies were chosen after a pilot phase where 20 participants answered 50 analogies and were scored for accuracy. The most challenging 10 analogies were removed and the remaining 40 were solved by participants with an accuracy of 65% (*SD* = 22%). These 40 analogies were divided into two lists of 20. A list of all analogies used in the experiment is available in the Supplementary Material.

#### Procedure

Participants were presented with the experiment on Psyscope – a graphical user interface (GUI) program used to develop psychology experiments (Cohen, 1993). Before beginning the experiment, participants were provided with both verbal and written instructions that indicated they would be completing a verbal analogy and memory task. Participants were shown an example analogy with its solution and completed one example trial of the experiment. Participants were also informed that they would receive instructions on how to position their hands during different phases in the experiment.

After receiving the instructions, participants were presented with the first of two counterbalanced blocks. In each block, participants were presented with an analogy and were given unlimited time to solve it. Once participants solved the analogy, they pressed a button and were presented with a list of six pseudorandom numbers for the memory task. After viewing the numbers for 5 s, the original analogy returned to the screen and participants explained how they arrived at their answer. After finishing their explanation, participants were asked to recall the six numbers by writing them on a worksheet. The participants completed this process for 20 analogies in each of the two blocks.

Before each block participants were given instructions on how to position their hands. In previous research, multiple instructions have been used to encourage and discourage gesture use such as, permitting and not permitting movement (Goldin-Meadow et al., 2001) or directly asking participants to gesture (Cook et al., 2012) without a change in results. We chose to explicitly instruct participants to gesture to increase the likelihood of gesture production in the gesture encouraged condition. For the gesture prohibited instructions, participants were instructed to keep their hands flat and still on the table in front of them. If the experimenter noticed a participant not following the gesture instructions, they gently reminded the participants to keep their hands still or gesture as needed. Instructions and blocks were counterbalanced such that the order and pairing of gesture instruction and analogy list occurred in all possible combinations across participants. Participants completed the entirety of the experiment in under an hour.

### Results and Discussion

A paired-samples *t*-test was conducted to compare performance on the secondary memory task in the gesture encouraged and gesture prohibited conditions. Results showed that participants remembered more digits when being prohibited from gesturing (*M* = 0.42, *SE* = 0.3) than being encouraged to gesture (*M* = 0.39, *SE* = 0.03), *t*(39) = 2.15, *p* = 0.04, *d* = 0.34. These results demonstrate a reversal in previous findings (e.g., Goldin-Meadow et al., 2001; Wagner et al., 2004; Cook et al., 2012) – producing gestures resulted in worse performance on the secondary memory task than being prohibited from gesturing. This indicates that gestures produced while explaining verbal analogies may not free up resources in working memory.

We conducted several follow-up analyses to assess the influence of order of gesture instructions and item effects. First, we evaluated whether the order of the two instruction conditions (gesture encouraged and gesture prohibited) influenced recall. A repeated measures ANOVA with gesture instruction as a within-subjects factor and order of conditions as a between-subjects factor revealed a main effect of gesture instruction [*F*(1,38) = 4.93, *p* = 0.03, *η* ^2^ = 0.12], but no main effect of order [*F*(1,38) = 0.51, *p* = 0.48, *η* ^2^ = 0.01] or interaction between order and gesture instruction [*F*(1,38) = 2.36, *p* = 0.13, *η* ^2^ = 0.06]. This indicates that irrespective the order of instructions, being instructed to gesture resulted in lower performance on digit recall compared to instructions to not gesture.

A univariate general linear model (GLM) analysis was conducted to examine whether the influence of gesture instruction on recall persists when controlling for variability from the analogy items and differences across participants. The model included gesture instruction as a fixed factor, and analogy item (nested within gesture instruction) and participant as random factors. The GLM analysis revealed significant main effects of gesture instruction (*F* = 5.09, *p* = 0.027) and participant (*F* = 10.01, *p* > 0.01), but not analogy (*F* = 1.28, *p* = 0.054). A summary of the analysis is available in Table 1. These results show that participants performed better on the memory task when prohibited from gesturing rather than being encouraged to gesture, even when controlling for item and participant variability.

**Table 1:** Univariate general linear model (GLM) analysis between gesture instruction, analogy, and participant on recall.

	*df*	*F*	*p*
Gesture instruction	1	5.09	0.027
Participant	38	10.01	>0.01
Analogy	77	1.28	0.054

The model considered gesture instruction as a fixed factor and participant and analogy as random factors.

Perhaps some analogies in this study were better suited to gesturing than others, and that gesture only reduces working memory load for concepts that are readily gestured about. Although people produce gestures while speaking about all kinds of information, previous research has shown that gestures are produced more frequently and consistently for speech that has content related to visuospatial information (Krauss, 1998; Alibali et al., 2001). Theories, such as the gesture as simulated action framework (Hostetter and Alibali, 2008, 2019), further argue that gestures are produced in part as result of visuospatial simulations. It is possible then that only analogies that depict visuospatial relationships would show a benefit of gesturing during explanations for working memory. To examine this possibility, we divided the analogies into two categories: analogies which focused on spatial relationships and shapes (e.g., belt is to waist as equator is to? and kite is to diamond as egg is to?) and analogies unrelated to special relationships such as those about color (e.g., apple is to banana as red is to?) and conducted a repeated measures ANOVA with gesture instructions (gesture encouraged and gesture prohibited) and analogy type (spatial and other) as within-subjects variables. The analysis revealed a significant main effect of gesture instruction [*F*(1, 38) = 5.14, *p* = 0.029, *η* ^2^ = 0.12], but no main effect of analogy type [*F*(1, 38) = 0.003, *p* = 0.95, *η* ^2^ > 0.00] or interaction between gesture instruction and analogy type [*F*(1, 38) = 2.29, *p* = 0.014, *η* ^2^ = 0.06]. These results indicated that irrespective of the type of analogy, participants performed better on the secondary memory task when being prohibited from gesture rather than being encouraged to gesture.

## Experiment 1B

Experiment 1a showed that unlike previous research where gesture production has been shown to help manage working memory load (e.g., Goldin-Meadow et al., 2001), being instructed to gesture during the explanation of verbal analogies did not help reduce working memory load and instead led to worse performance when compared to being prohibited from gesturing. Given this surprising finding, Experiment 1b was conducted to replicate the main finding of Experiment 1a. Additionally, Experiment 1b employed a few small changes to eliminate potential participant fatigue, distraction, and more closely align with the methods used in previous research.

### Method

#### Participants

Twenty-one undergraduates from UCSC participated for partial course credit. Participants were recruited from psychology courses at UCSC through the Sona Systems subject pool and were required to be native speakers of English to be eligible for the study. The study was reviewed and approved by the UCSC and participants provided their written informed consent to participate in this study.

#### Materials

Materials consisted of a subset of analogies from Experiment 1a. A full list is available in the Supplementary Material.

#### Procedure

The procedure was the same as Experiment 1a with few minor changes. Participants were fewer verbal analogies (15 in each condition) to eliminate potential effects of fatigue. Additionally, the secondary memory task was changed to pseudorandom consonants (e.g., v, r, k, p, q, and d) instead of numbers to match the task used by Goldin-Meadow et al. (2001). Finally, participants entered their responses to the secondary task with the keyboard instead of on paper to reduce potential costs of switching between using the computer and paper.

### Results and Discussion

Experiment 1b showed the same pattern of results as Experiment 1a, with participants recalling more consonants when instructed to prohibit gesture (*M* = 0.38, *SE* = 0.4) rather than produce gestures (*M* = 0.33, *SE* = 0.04), *t*(20) = 2.16, *p* = 0.04, *d* = 0.47. A repeated measures ANOVA with instruction order as a between-subjects factor found a main effect of gesture instruction [*F*(1,19) = 4.52, *p* = 0.047, *η*^2^ = 0.192] but no effect of order [*F*(1,19) = 0.49, *p* = 0.49, *η*^2^ = 0.03] and no interaction between order and instruction [*F*(1,19) = 1.40, *p* = 0.25, *η*^2^ = 0.07]. A summary of the results of both experiments can be seen in Table 2.

**Table 2:** Mean proportion recalled for Experiments 1a and 1b.

Gesture instruction		
Experiments	Gesture encouraged	Gesture prohibited
Experiment 1a	0.39 (0.03)	0.42 (0.03)
Experiment 1b	0.33 (0.04)	0.38 (0.04)

Standard error in parentheses.

## General Discussion

Gesturing during explanations has previously been shown to alleviate working memory load (Goldin-Meadow et al., 2001; Wagner et al., 2004; Cook et al., 2012). These studies have used dual-task paradigms to show that when individuals gesture during an explanation task their performance on a secondary memory task is enhanced compared to explaining without using gestures. These findings demonstrate that a speaker’s own gestures can influence their working memory and allow speakers to allocate cognitive resources that would have otherwise been used in an explanation to a secondary task.

The aim of the present research was to build on the previous research of Goldin-Meadow et al. (2001), Wagner et al. (2004), Cook et al. (2012), and others to explore whether gesture during a novel verbal analogy task would have similar effects on working memory load. Unlike previous research, our studies showed that gesturing during the explanation of verbal analogies did not lighten working memory load. Instead, being instructed to gesture led to worse performance on a secondary memory task when compared to being prohibited from gesturing. These results suggested that although producing gestures may help manage working memory load in some contexts, it may create additional load in working memory in other contexts.

There are several possible explanations for why gesture did not reduce working memory load during the explanation of analogies. For one, explaining and gesturing about verbal analogies may have led participants to use cognitive resources to build visuospatial representations of the content of the analogies. According to the gesture as simulated action framework (Hostetter and Alibali, 2008, 2019), gestures emerge from embodied visuospatial and motor representations used in speaking and thinking. For an example, consider solving the analogy, “belt is to waist, as equator is to …” In the explanation, a participant could explain that belts go around the waist, as an equator goes around the globe and produce a gesture of one hand circling around another or around the participant’s body. The “going around” gesture is an emergent action of the motor representation of the visuospatial concept “going around” that is needed to solve the analogy problem. Creating and maintaining this representation in mind in service of producing a gesture could add more load than being prohibited from gesture and not needing to construct such vivid visuospatial representations.

Another possibility is that the different ways participants recruit gestures in their explanations could have varying effects on working memory. In explaining an analogy, gestures could be used to highlight different relationships that are key for solving the analogy that differ across problems, index words on the screen, or provide rhythm to the explanatory speech. These usages of gesture may inconsistently interact with working memory load with some gestures being more effective than others and freeing up cognitive resources. While we do not have video data to explore these possibilities in the present study, investigating the association between the gesture strategy used by participants and extent of working memory load could help clarify these relationships in future research.

The difficulty and unfamiliarity of analogies may have also influenced the results of our studies. Verbal analogies are not typically taught in schooling and participants may have had little experience solving and providing explanations for analogies. Our analogies were more challenging for participants than factoring problems used in previous research and participants did not have a means to “check their answers” to see if they reached the correct solution. The potential difficulty and unfamiliarity with the task could have created additional load for participants and influenced results. However, since the analogies and gesture instruction condition were counterbalanced across participants in present study, we believe that factors such as difficulty alone cannot explain the observed difference between the gesture encouraged and gesture prohibited conditions.

There are several limitations to consider when interpreting the results of this research. First, our manipulation consisted of instructing participants to produce or prohibit gestures. Although this gave us a clear comparison between two conditions, it also removed the possibility of determining a baseline rate of gesture for each of our participants. It is possible that individuals who gesture more in their day-to-day lives may differentially benefit from gesture use than individuals who gesture infrequently. Encouraging our participants to gesture may have also been more distracting from the secondary memory task than prohibiting them from gesturing. Differences in recall could have been due to the added difficulty of remembering to produce gestures which may have been greater than the difficulty of inhibiting gesture. Additionally, the absence of video data limits our ability to compare our results with previous studies and analyze how specific gestures and the consistency of the gestures produced may have influenced offloading. Follow-up research could elaborate on the verbal analogies themselves by developing analogies that have been evaluated for reliability and investigating how gesture use influences the accuracy of solving the analogies and memory for the solutions. Future research can also examine the background of participants and consider whether age or other demographic factors influence the results.

These findings highlight the need for a nuanced approach to studying the relationship between gesture and other cognitive processes. While the gestures produced in the explanation of mathematical and other types of problems may save cognitive resources, those produced during the explanation of analogies may have the opposite effect. Similarly, although gesture can aid in problem solving in some domains (such as mental rotation), it can bias a problem solver and lead to less efficient or incorrect problem solving in other scenarios (Alibali et al., 2011; Göksun et al., 2013; Hostetter et al., 2016). Both the gestures and the context in which they are produced may influence the extent to which gesture is beneficial to a speaker.

The results of this initial work on verbal analogies and gesture indicates that the content gestures refer to may matter in its relationship to working memory load. Gestures may not interact with all spoken content equally, but instead may adapt to the constraints of a situation. This work adds to a growing literature that demonstrates context, individual differences, and type of gesture influence how gesture production interacts with cognition.

## Data Availability Statement

The datasets presented in this study can be found in online repositories. The names of the repository/repositories and accession number(s) can be found below: https://osf.io/bja5c/?view_only=0bdd50a2aa664e45ada5b4b7a0b807bf – Open Science Framework.

## Ethics Statement

The studies involving human participants were reviewed and approved by University of California, Santa Cruz Institutional Review Board. The patients/participants provided their written informed consent to participate in this study.

## Author Contributions

AO and MW contributed to conception and design of the study. AO organized and analyzed the data and wrote the initial draft of the manuscript. MW provided feedback on the manuscript and approved of a final version that was completed by AO. All authors contributed to the article and approved the submitted version.

### Conflict of Interest

The authors declare that the research was conducted in the absence of any commercial or financial relationships that could be construed as a potential conflict of interest.
