# Supplementary material for: Does Gesture Lighten the Load? The Case of Verbal Analogies
Source: Front Psychol. 2020 Sep 17;11:571109. doi: 10.3389/fpsyg.2020.571109 (PMC7528622; doi:10.3389/fpsyg.2020.571109)
Supplement: Supplementary file 1 [file Data_Sheet_1.pdf]

Experiment 1a Analogies

Apple is to banana as red is to (yellow)

Stop sign is to yield sign as octagon is to (triangle)

Kite is to diamond as egg is to (oval)

Balls are to dice as spheres are to (cubes)

Sandpaper is to silk as razor stubble is to (clean-shaven)

Cardboard is to glass as opaque is to (clear)

Crumble is to brittle as stretch is to (flexible)

Rectangle is to box as triangle is to (pyramid)

Carrot is to eggplant as tangerine is to (plum)

Strawberry is to kiwi as radish is to (celery)

Cup is to coffee as bowl is to (soup)

Belt is to waist as equator is to (earth)

Wrapping is to present as clothes are to (body)

Water is to bottle as blood is to (veins)

Freeway is to parking lot as river is to (lake, ocean)

Bulls-eye is to target as downtown is to (city)

Orange is to peel as pie is to (crust)

Roller-coaster is to train as wiggly is to (straight)

Window is to pane as book is to (page)

Pulling is to grabbing as pushing is to (throwing)

Circle is to oval as square is to (rectangle)

Spotted is to striped as cheetah is to (tiger)

Robin is to blue jay as raspberry is to (blueberry)

Brick is to rock candy as pillow is to (marshmallow)

Football is to basketball as lemon is to (orange)

Sand is to glass as coal is to (diamond)

Water is to fluid as honey is to (sticky)

Picture is to blurred as knife is to (dull)

Mustard is to pickle relish as lemons are to (limes)

Metal is to wool as slick is to (fuzzy)

Racecar is to slow vehicle as run is to (walk)

Hat is to head as roof is to (house)

Electrons are to nucleus as planets are to (sun)

Chest is to treasure as egg is to (yolk)

Crumble is to cookie as rubble is to (building)

Rectangle is to perimeter as nation is to (border)

Football field is to 50-yard-line as tennis court is to (net)

Treadmill is to ladder as run is to (climb)

Sliding is to skiing as climbing is to (hiking, rock climbing)

Lightning is to electrical cord as flood is to (stream/water pipe)

Experiment 1b Analogies

Rectangle is to box as triangle is to (pyramid)

Wrapping is to present as clothes are to (body)

Belt is to waist as equator is to (earth)

Kite is to diamond as egg is to (oval)

Window is to pane as book is to (page)

Balls are to dice as spheres are to (cubes)

Orange is to peel as pie is to (crust)

Bulls-eye is to target as downtown is to (city)

Freeway is to parking lot as river is to (lake, ocean)

Crumble is to brittle as stretch is to (flexible)

Water is to bottle as blood is to (veins)

Cup is to coffee as bowl is to (soup)

Apple is to banana as red is to (yellow)

Pulling is to grabbing as pushing is to (throwing)

Stop sign is to yield sign as octagon is to (triangle)

Football is to basketball as lemon is to (orange)

Rectangle is to perimeter as nation is to (border)

Chest is to treasure as egg is to (yolk)

Picture is to blurred as knife is to (dull)

Electrons are to nucleus as planets are to (sun)

Spotted is to striped as cheetah is to (tiger)

Robin is to blue jay as raspberry is to (blueberry)

Treadmill is to ladder as run is to (climb)

Brick is to rock candy as pillow is to (marshmallow)

Football field is to 50-yard-line as tennis court is to (net)

Sand is to glass as coal is to (diamond)

Crumble is to cookie as rubble is to (building)

Hat is to head as roof is to (house)

Racecar is to slow vehicle as run is to (walk)

Circle is to oval as square is to (rectangle)
